# {μ_2_-1,4-Bis[2-(4-pyrid­yl)ethen­yl]benzene-κ^2^ *N*:*N*′}bis­[bis­(acetyl­acetonato-κ^2^ *O*,*O*′)copper(II)]

**DOI:** 10.1107/S1600536809048582

**Published:** 2009-11-21

**Authors:** Fang-Fang Jian, Jing Wang, Jing Zhang

**Affiliations:** a Microscale Science Institute, Weifang University, Weifang 261061, People’s Republic of China; b New Materials and Function Coordination Chemistry Laboratory, Qingdao University of Science and Technology, Qingdao 266042, People’s Republic of China

## Abstract

The asymmetric unit of the title compound, [Cu_2_(C_5_H_7_O_2_)_4_(C_20_H_16_N_2_)], contains half of a centrosymmetric dinuclear mol­ecule. In the mol­ecule, each Cu center is coordinated by four O atoms from two acetyl­acetonate ligands and one N atom from the bridging linear 1,4-bis­[2-(4-pyrid­yl)ethen­yl]benzene ligand in a square-pyramidal geometry. In the crystal structure, weak inter­molecular C—H⋯O hydrogen bonds link mol­ecules into sheets parallel to the *bc* plane.

## Related literature

For coordination complexes with inter­esting topologies or properties, see: Ma *et al.* (2009 ▶); Liu *et al.* (2008 ▶). For long ligands, see: Banfi *et al.* (2002 ▶); Niu *et al.* (2001 ▶); Coe *et al.* (2006 ▶).
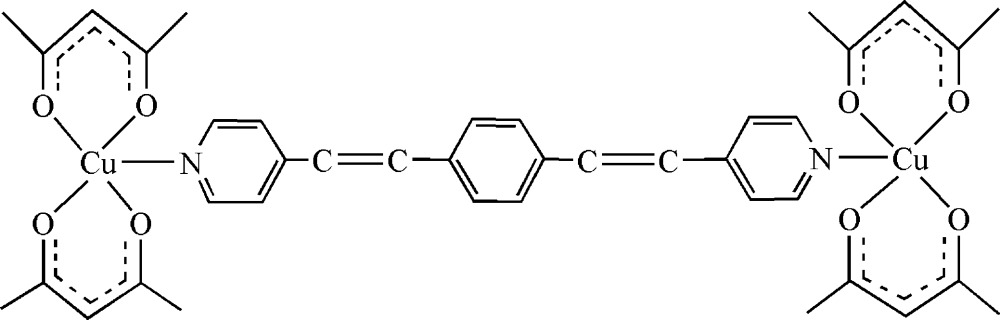


## Experimental

### 

#### Crystal data

[Cu_2_(C_5_H_7_O_2_)_4_(C_20_H_16_N_2_)]
*M* *_r_* = 807.85
Monoclinic, 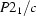

*a* = 7.9584 (16) Å
*b* = 18.594 (4) Å
*c* = 15.063 (4) Å
β = 120.97 (2)°
*V* = 1911.2 (8) Å^3^
*Z* = 2
Mo *K*α radiation
μ = 1.17 mm^−1^
*T* = 293 K
0.25 × 0.21 × 0.20 mm

#### Data collection

Bruker SMART CCD area-detector diffractometer
Absorption correction: multi-scan (*SADABS*; Sheldrick, 2000 ▶) *T*_min_ = 0.759, *T*_max_ = 0.800
7784 measured reflections
3352 independent reflections
2807 reflections with *I* > 2σ(*I*)
*R*_int_ = 0.022

#### Refinement

*R*[*F* ^2^ > 2σ(*F* ^2^)] = 0.043
*wR*(*F* ^2^) = 0.113
*S* = 0.99
3352 reflections
235 parameters
H-atom parameters constrained
Δρ_max_ = 0.41 e Å^−3^
Δρ_min_ = −0.19 e Å^−3^

### 

Data collection: *SMART* (Bruker, 1997 ▶); cell refinement: *SAINT* (Bruker, 1997 ▶); data reduction: *SAINT*; program(s) used to solve structure: *SHELXS97* (Sheldrick, 2008 ▶); program(s) used to refine structure: *SHELXL97* (Sheldrick, 2008 ▶); molecular graphics: *SHELXTL* (Sheldrick, 2008 ▶); software used to prepare material for publication: *SHELXTL*.

## Supplementary Material

Crystal structure: contains datablocks global, I. DOI: 10.1107/S1600536809048582/cv2655sup1.cif

Structure factors: contains datablocks I. DOI: 10.1107/S1600536809048582/cv2655Isup2.hkl

Additional supplementary materials:  crystallographic information; 3D view; checkCIF report

## Figures and Tables

**Table 1:** Hydrogen-bond geometry (Å, °)

*D*—H⋯*A*	*D*—H	H⋯*A*	*D*⋯*A*	*D*—H⋯*A*
C14—H14*A*⋯O2^i^	0.93	2.45	3.371 (4)	173
C16—H16*A*⋯O1^ii^	0.93	2.52	3.333 (4)	147
C16—H16*A*⋯O3^ii^	0.93	2.58	3.315 (4)	136

Symmetry codes: (i) 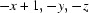; (ii) 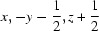.

**Fig. 1.**
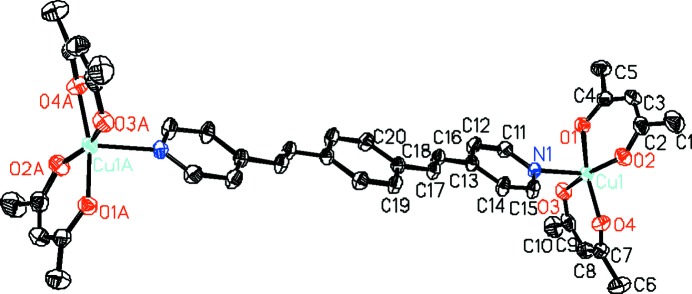
The molecular structure of the title compound with the atom-labeling scheme [symmetry code: (A) 1-x, -y, 1-z]. H atoms omited for clarity. Displacement ellipsoids are drawn at the 30% probability level.

## supplementary crystallographic information

### Comment

Metal ions and organic ligands are considered as the most important factors for designing the coordination networks (Ma *et al.*, 2009). Up to now, it is still a challenge to predict the exact structure and understand the roles of both factors in crystal engineering. The flexible bridging ligands can afford different conformation with interesting topologies or properties (Liu *et al.*, 2008). Among the others, long bis(pyridyl) ligands are used to construct the connectivity and geometry with different coordination sites metal ions, and often lead to interesting structural motifs (Ma *et al.*, 2009). A large number of examples of particularly long ligands - 1,4-phenylenebis(4-pyridylmethanone), bis(4-pyridyl)terephthalate (Banfi *et al.*, 2002), *N*,*N*^'^-bis(4-pyridylmethyl)piperazine (Niu *et al.*, 2001), *N*-phenyl-1,4-bis(*E*-2-(4-pyridyl)ethenyl)benzene (Coe *et al.*, 2006), have been adopted for the self-assembly of coordination polymers, such as one-dimensional coordination chains, double helices, two dimensional layered structures, interpenetrated ladders, interpenetrated frameworks and so on (Banfi *et al.*, 2002). Herein, we present the shoulder-pole coordination compound based on 1,4-bis(2-(4-pyridyl)ethenyl)benzole (bpyph) ligand, and describe its crystal structure.

In the title structure (Fig. 1),each Cu center is coordinated by four O atoms and one N atom from the bpyph ligand in a distorted pyramidal geometry. The linear bpyph ligand links two acetylacetonate copper(II) by Cu—N bonds, displaying the shoulder-pole model. In the crystal structure, weak intermolecular C—H···O hydrogen bonds (Table 1) link molecules into sheets parallel to *bc* plane.

### Experimental

1,4-Bis(2-(4-pyridyl)ethenyl)benzene (2.84 g, 0.01 mol) and acetylacetonate copper(II) (5.23 g, 0.02 mol) in 2:1 molar ratio was dissolved in ethanol solution (40 ml) and refluxed for 2 h. After cooling and filtering, the blue block crystals were collected after 4 days (yield 42.35%).

### Refinement

All H atoms were positioned geometrically (C—H 0.93–0.96 Å) and allowed to ride on their parent atoms, with *U*_iso_(H) = 1.2*U*_eq_ of the parent atom.

### Crystal data

**Table:**

[Cu_2_(C_5_H_7_O_2_)_4_(C_20_H_16_N_2_)]	*F*(000) = 840
*M**_r_* = 807.85	*D*_x_ = 1.404 Mg m^−^^3^
Monoclinic, *P*2_1_/*c*	Mo *K*α radiation, λ = 0.71073 Å
Hall symbol: -P 2ybc	Cell parameters from 1253 reflections
*a* = 7.9584 (16) Å	θ = 1.9–25.0°
*b* = 18.594 (4) Å	µ = 1.17 mm^−^^1^
*c* = 15.063 (4) Å	*T* = 293 K
β = 120.97 (2)°	Block, blue
*V* = 1911.2 (8) Å^3^	0.25 × 0.21 × 0.20 mm
*Z* = 2	

### Data collection

**Table:**

Bruker SMART CCD area-detector diffractometer	3352 independent reflections
Radiation source: fine-focus sealed tube	2807 reflections with *I* > 2σ(*I*)
graphite	*R*_int_ = 0.022
phi and ω scans	θ_max_ = 25.0°, θ_min_ = 1.9°
Absorption correction: multi-scan (*SADABS*; Sheldrick, 2000)	*h* = −9→5
*T*_min_ = 0.759, *T*_max_ = 0.800	*k* = −22→20
7784 measured reflections	*l* = −17→17

### Refinement

**Table:**

Refinement on *F*^2^	Primary atom site location: structure-invariant direct methods
Least-squares matrix: full	Secondary atom site location: difference Fourier map
*R*[*F*^2^ > 2σ(*F*^2^)] = 0.043	Hydrogen site location: inferred from neighbouring sites
*wR*(*F*^2^) = 0.113	H-atom parameters constrained
*S* = 0.99	*w* = 1/[σ^2^(*F*_o_^2^) + (0.0573*P*)^2^ + 1.3432*P*] where *P* = (*F*_o_^2^ + 2*F*_c_^2^)/3
3352 reflections	(Δ/σ)_max_ < 0.001
235 parameters	Δρ_max_ = 0.41 e Å^−^^3^
0 restraints	Δρ_min_ = −0.19 e Å^−^^3^

### Special details

**Table:**

Geometry. All e.s.d.'s (except the e.s.d. in the dihedral angle between two l.s. planes) are estimated using the full covariance matrix. The cell e.s.d.'s are taken into account individually in the estimation of e.s.d.'s in distances, angles and torsion angles; correlations between e.s.d.'s in cell parameters are only used when they are defined by crystal symmetry. An approximate (isotropic) treatment of cell e.s.d.'s is used for estimating e.s.d.'s involving l.s. planes.
Refinement. Refinement of *F*^2^ against ALL reflections. The weighted *R*-factor *wR* and goodness of fit *S* are based on *F*^2^, conventional *R*-factors *R* are based on *F*, with *F* set to zero for negative *F*^2^. The threshold expression of *F*^2^ > σ(*F*^2^) is used only for calculating *R*-factors(gt) *etc*. and is not relevant to the choice of reflections for refinement. *R*-factors based on *F*^2^ are statistically about twice as large as those based on *F*, and *R*- factors based on ALL data will be even larger.

### Fractional atomic coordinates and isotropic or equivalent isotropic displacement parameters (Å^2^)

**Table:**

	*x*	*y*	*z*	*U*_iso_*/*U*_eq_	
Cu1	0.51253 (5)	−0.173135 (19)	−0.13916 (3)	0.04299 (16)	
O1	0.3204 (3)	−0.25040 (12)	−0.18542 (17)	0.0531 (6)	
O2	0.3110 (3)	−0.10340 (12)	−0.22282 (18)	0.0586 (6)	
O3	0.7113 (3)	−0.24740 (12)	−0.09329 (17)	0.0564 (6)	
O4	0.6960 (3)	−0.09862 (12)	−0.12417 (19)	0.0604 (6)	
N1	0.5307 (4)	−0.15375 (13)	0.01154 (18)	0.0414 (6)	
C1	0.0211 (6)	−0.0532 (3)	−0.3578 (4)	0.0995 (16)	
H1A	0.1015	−0.0110	−0.3323	0.149*	
H1B	−0.0934	−0.0470	−0.3527	0.149*	
H1C	−0.0177	−0.0609	−0.4288	0.149*	
C2	0.1362 (5)	−0.1177 (2)	−0.2939 (3)	0.0629 (9)	
C3	0.0540 (5)	−0.1850 (2)	−0.3146 (3)	0.0691 (11)	
H3A	−0.0755	−0.1890	−0.3684	0.083*	
C4	0.1463 (5)	−0.2471 (2)	−0.2628 (3)	0.0579 (9)	
C5	0.0410 (7)	−0.3181 (2)	−0.2979 (4)	0.0868 (14)	
H5A	0.1251	−0.3558	−0.2540	0.130*	
H5B	0.0066	−0.3271	−0.3681	0.130*	
H5C	−0.0757	−0.3165	−0.2942	0.130*	
C6	0.9651 (7)	−0.0436 (3)	−0.1201 (4)	0.1045 (17)	
H6A	0.8839	−0.0022	−0.1323	0.157*	
H6B	0.9885	−0.0490	−0.1763	0.157*	
H6C	1.0878	−0.0374	−0.0565	0.157*	
C7	0.8632 (6)	−0.1098 (2)	−0.1128 (3)	0.0648 (10)	
C8	0.9518 (6)	−0.1755 (2)	−0.0957 (3)	0.0747 (12)	
H8A	1.0737	−0.1769	−0.0901	0.090*	
C9	0.8778 (5)	−0.2392 (2)	−0.0861 (3)	0.0623 (10)	
C10	0.9943 (7)	−0.3073 (3)	−0.0657 (4)	0.0896 (14)	
H10A	0.9217	−0.3470	−0.0616	0.134*	
H10B	1.1167	−0.3029	−0.0015	0.134*	
H10C	1.0187	−0.3154	−0.1210	0.134*	
C11	0.4967 (5)	−0.20205 (18)	0.0647 (2)	0.0505 (8)	
H11A	0.4738	−0.2492	0.0409	0.061*	
C12	0.4930 (5)	−0.18686 (17)	0.1532 (2)	0.0519 (8)	
H12A	0.4707	−0.2235	0.1879	0.062*	
C13	0.5224 (4)	−0.11714 (16)	0.1905 (2)	0.0434 (7)	
C14	0.5614 (5)	−0.06650 (17)	0.1356 (2)	0.0514 (8)	
H14A	0.5842	−0.0188	0.1573	0.062*	
C15	0.5661 (5)	−0.08703 (17)	0.0498 (2)	0.0513 (8)	
H15A	0.5959	−0.0522	0.0157	0.062*	
C16	0.5141 (5)	−0.10005 (17)	0.2825 (2)	0.0488 (8)	
H16A	0.5061	−0.1388	0.3192	0.059*	
C17	0.5167 (5)	−0.03530 (16)	0.3193 (2)	0.0451 (7)	
H17A	0.5261	0.0034	0.2830	0.054*	
C18	0.5065 (4)	−0.01843 (16)	0.4102 (2)	0.0420 (7)	
C19	0.5343 (5)	0.05182 (16)	0.4469 (2)	0.0501 (8)	
H19A	0.5577	0.0877	0.4115	0.060*	
C20	0.4717 (5)	−0.07005 (17)	0.4662 (2)	0.0505 (8)	
H20A	0.4522	−0.1177	0.4444	0.061*	

### Atomic displacement parameters (Å^2^)

**Table:**

	*U*^11^	*U*^22^	*U*^33^	*U*^12^	*U*^13^	*U*^23^
Cu1	0.0456 (2)	0.0468 (2)	0.0388 (2)	0.00112 (16)	0.02331 (18)	−0.00511 (16)
O1	0.0523 (14)	0.0536 (13)	0.0514 (13)	−0.0059 (10)	0.0252 (11)	−0.0089 (10)
O2	0.0541 (15)	0.0537 (14)	0.0549 (14)	0.0064 (11)	0.0186 (12)	−0.0004 (11)
O3	0.0531 (14)	0.0571 (14)	0.0565 (14)	0.0087 (11)	0.0263 (12)	−0.0045 (11)
O4	0.0600 (15)	0.0584 (14)	0.0720 (16)	−0.0050 (11)	0.0405 (13)	−0.0016 (12)
N1	0.0477 (15)	0.0439 (14)	0.0347 (13)	0.0026 (11)	0.0226 (11)	−0.0037 (10)
C1	0.073 (3)	0.101 (4)	0.094 (4)	0.027 (3)	0.021 (3)	0.024 (3)
C2	0.054 (2)	0.080 (3)	0.052 (2)	0.0147 (19)	0.0260 (18)	0.0080 (18)
C3	0.045 (2)	0.093 (3)	0.055 (2)	−0.006 (2)	0.0155 (17)	0.003 (2)
C4	0.055 (2)	0.077 (2)	0.049 (2)	−0.0143 (18)	0.0314 (18)	−0.0138 (18)
C5	0.076 (3)	0.094 (3)	0.084 (3)	−0.035 (2)	0.037 (3)	−0.021 (2)
C6	0.088 (3)	0.118 (4)	0.122 (4)	−0.030 (3)	0.065 (3)	0.000 (3)
C7	0.059 (2)	0.089 (3)	0.053 (2)	−0.012 (2)	0.0331 (19)	−0.0033 (19)
C8	0.050 (2)	0.103 (3)	0.078 (3)	0.007 (2)	0.038 (2)	0.008 (2)
C9	0.053 (2)	0.089 (3)	0.0406 (18)	0.019 (2)	0.0211 (17)	−0.0028 (18)
C10	0.077 (3)	0.110 (4)	0.084 (3)	0.037 (3)	0.043 (3)	0.008 (3)
C11	0.064 (2)	0.0442 (17)	0.0515 (19)	−0.0086 (15)	0.0354 (17)	−0.0137 (15)
C12	0.076 (2)	0.0423 (17)	0.052 (2)	−0.0084 (15)	0.0434 (19)	−0.0021 (14)
C13	0.0547 (19)	0.0422 (16)	0.0375 (16)	0.0041 (14)	0.0269 (14)	−0.0002 (13)
C14	0.083 (2)	0.0383 (17)	0.0457 (18)	0.0030 (16)	0.0419 (18)	−0.0020 (13)
C15	0.076 (2)	0.0444 (18)	0.0431 (17)	0.0049 (16)	0.0371 (17)	0.0047 (14)
C16	0.071 (2)	0.0450 (18)	0.0416 (17)	0.0018 (15)	0.0368 (16)	0.0023 (13)
C17	0.061 (2)	0.0444 (17)	0.0392 (16)	0.0014 (14)	0.0329 (15)	0.0028 (13)
C18	0.0495 (18)	0.0436 (16)	0.0356 (15)	0.0021 (13)	0.0239 (14)	−0.0014 (13)
C19	0.075 (2)	0.0424 (17)	0.0457 (18)	−0.0034 (15)	0.0401 (17)	0.0014 (13)
C20	0.074 (2)	0.0399 (17)	0.0471 (18)	−0.0049 (15)	0.0379 (17)	−0.0075 (14)

### Geometric parameters (Å, °)

**Table:**

Cu1—O2	1.939 (2)	C7—C8	1.367 (5)
Cu1—O4	1.940 (2)	C8—C9	1.363 (6)
Cu1—O3	1.940 (2)	C8—H8A	0.9300
Cu1—O1	1.947 (2)	C9—C10	1.504 (5)
Cu1—N1	2.228 (2)	C10—H10A	0.9600
O1—C4	1.273 (4)	C10—H10B	0.9600
O2—C2	1.273 (4)	C10—H10C	0.9600
O3—C9	1.282 (4)	C11—C12	1.378 (4)
O4—C7	1.269 (4)	C11—H11A	0.9300
N1—C11	1.320 (4)	C12—C13	1.384 (4)
N1—C15	1.335 (4)	C12—H12A	0.9300
C1—C2	1.515 (5)	C13—C14	1.389 (4)
C1—H1A	0.9600	C13—C16	1.456 (4)
C1—H1B	0.9600	C14—C15	1.368 (4)
C1—H1C	0.9600	C14—H14A	0.9300
C2—C3	1.371 (5)	C15—H15A	0.9300
C3—C4	1.376 (5)	C16—C17	1.321 (4)
C3—H3A	0.9300	C16—H16A	0.9300
C4—C5	1.505 (5)	C17—C18	1.448 (4)
C5—H5A	0.9600	C17—H17A	0.9300
C5—H5B	0.9600	C18—C19	1.391 (4)
C5—H5C	0.9600	C18—C20	1.397 (4)
C6—C7	1.511 (6)	C19—C20^i^	1.376 (4)
C6—H6A	0.9600	C19—H19A	0.9300
C6—H6B	0.9600	C20—C19^i^	1.376 (4)
C6—H6C	0.9600	C20—H20A	0.9300
			
O2—Cu1—O4	85.37 (10)	O4—C7—C6	114.9 (4)
O2—Cu1—O3	163.25 (10)	C8—C7—C6	119.9 (4)
O4—Cu1—O3	92.29 (11)	C9—C8—C7	125.9 (4)
O2—Cu1—O1	91.51 (10)	C9—C8—H8A	117.0
O4—Cu1—O1	166.93 (10)	C7—C8—H8A	117.0
O3—Cu1—O1	87.05 (10)	O3—C9—C8	125.4 (3)
O2—Cu1—N1	98.77 (10)	O3—C9—C10	114.7 (4)
O4—Cu1—N1	96.62 (10)	C8—C9—C10	119.9 (4)
O3—Cu1—N1	97.98 (9)	C9—C10—H10A	109.5
O1—Cu1—N1	96.40 (9)	C9—C10—H10B	109.5
C4—O1—Cu1	125.2 (2)	H10A—C10—H10B	109.5
C2—O2—Cu1	125.9 (2)	C9—C10—H10C	109.5
C9—O3—Cu1	124.3 (2)	H10A—C10—H10C	109.5
C7—O4—Cu1	124.9 (2)	H10B—C10—H10C	109.5
C11—N1—C15	115.7 (3)	N1—C11—C12	124.1 (3)
C11—N1—Cu1	125.5 (2)	N1—C11—H11A	118.0
C15—N1—Cu1	118.6 (2)	C12—C11—H11A	118.0
C2—C1—H1A	109.5	C11—C12—C13	120.1 (3)
C2—C1—H1B	109.5	C11—C12—H12A	120.0
H1A—C1—H1B	109.5	C13—C12—H12A	120.0
C2—C1—H1C	109.5	C12—C13—C14	115.9 (3)
H1A—C1—H1C	109.5	C12—C13—C16	120.6 (3)
H1B—C1—H1C	109.5	C14—C13—C16	123.5 (3)
O2—C2—C3	124.7 (3)	C15—C14—C13	119.8 (3)
O2—C2—C1	114.2 (4)	C15—C14—H14A	120.1
C3—C2—C1	121.1 (4)	C13—C14—H14A	120.1
C2—C3—C4	125.7 (3)	N1—C15—C14	124.4 (3)
C2—C3—H3A	117.1	N1—C15—H15A	117.8
C4—C3—H3A	117.1	C14—C15—H15A	117.8
O1—C4—C3	124.8 (3)	C17—C16—C13	126.8 (3)
O1—C4—C5	115.3 (3)	C17—C16—H16A	116.6
C3—C4—C5	119.9 (3)	C13—C16—H16A	116.6
C4—C5—H5A	109.5	C16—C17—C18	126.7 (3)
C4—C5—H5B	109.5	C16—C17—H17A	116.6
H5A—C5—H5B	109.5	C18—C17—H17A	116.6
C4—C5—H5C	109.5	C19—C18—C20	116.5 (3)
H5A—C5—H5C	109.5	C19—C18—C17	120.3 (3)
H5B—C5—H5C	109.5	C20—C18—C17	123.2 (3)
C7—C6—H6A	109.5	C20^i^—C19—C18	122.2 (3)
C7—C6—H6B	109.5	C20^i^—C19—H19A	118.9
H6A—C6—H6B	109.5	C18—C19—H19A	118.9
C7—C6—H6C	109.5	C19^i^—C20—C18	121.3 (3)
H6A—C6—H6C	109.5	C19^i^—C20—H20A	119.3
H6B—C6—H6C	109.5	C18—C20—H20A	119.3
O4—C7—C8	125.2 (4)		

Symmetry codes: (i) −*x*+1, −*y*, −*z*+1.

### Hydrogen-bond geometry (Å, °)

**Table:**

*D*—H···*A*	*D*—H	H···*A*	*D*···*A*	*D*—H···*A*
C14—H14A···O2^ii^	0.93	2.45	3.371 (4)	173
C16—H16A···O1^iii^	0.93	2.52	3.333 (4)	147
C16—H16A···O3^iii^	0.93	2.58	3.315 (4)	136

Symmetry codes: (ii) −*x*+1, −*y*, −*z*; (iii) *x*, −*y*−1/2, *z*+1/2.
